# Enhancing Food Intake Tracking in Long-term Care With Automated Food Imaging and Nutrient Intake Tracking (AFINI-T) Technology: Validation and Feasibility Assessment

**DOI:** 10.2196/37590

**Published:** 2022-11-17

**Authors:** Kaylen Pfisterer, Robert Amelard, Jennifer Boger, Heather Keller, Audrey Chung, Alexander Wong

**Affiliations:** 1 Department of Systems Design Engineering University of Waterloo Waterloo, ON Canada; 2 Waterloo AI Institute Waterloo, ON Canada; 3 Schlegel-University of Waterloo Institute for Aging Waterloo, ON Canada; 4 Centre for Digital Therapeutics Techna Institute University Health Network Toronto, ON Canada; 5 KITE-Toronto Rehabilitation Institute University Health Network Toronto, ON Canada; 6 Department of Kinesiology and Health Sciences University of Waterloo Waterloo, ON Canada

**Keywords:** long-term care, automated nutrient intake, convolutional neural network, food segmentation, food classification, depth imaging, deep learning, collaborative design, aging, food intake

## Abstract

**Background:**

Half of long-term care (LTC) residents are malnourished, leading to increased hospitalization, mortality, and morbidity, with low quality of life. Current tracking methods are subjective and time-consuming.

**Objective:**

This paper presented the automated food imaging and nutrient intake tracking technology designed for LTC.

**Methods:**

A needs assessment was conducted with 21 participating staff across 12 LTC and retirement homes. We created 2 simulated LTC intake data sets comprising modified (664/1039, 63.91% plates) and regular (375/1039, 36.09% plates) texture foods. Overhead red-green-blue-depth images of plated foods were acquired, and foods were segmented using a pretrained food segmentation network. We trained a novel convolutional autoencoder food feature extractor network using an augmented UNIMIB2016 food data set. A meal-specific food classifier was appended to the feature extractor and tested on our simulated LTC food intake data sets. Food intake (percentage) was estimated as the differential volume between classified full portion and leftover plates.

**Results:**

The needs assessment yielded 13 nutrients of interest, requirement for objectivity and repeatability, and account for real-world environmental constraints. For 12 meal scenarios with up to 15 classes each, the top-1 classification accuracy was 88.9%, with mean intake error of −0.4 (SD 36.7) mL. Nutrient intake estimation by volume was strongly linearly correlated with nutrient estimates from mass (*r*^2^=0.92-0.99), with good agreement between methods (σ=−2.7 to −0.01; 0 within each of the limits of agreement).

**Conclusions:**

The automated food imaging and nutrient intake tracking approach is a deep learning–powered computational nutrient sensing system that appears to be feasible (validated accuracy against gold-standard weighed food method, positive end user engagement) and may provide a novel means for more accurate and objective tracking of LTC residents’ food intake to support and prevent malnutrition tracking strategies.

## Introduction

### Background

Malnutrition leads to high morbidity [1] and low quality of life [2]. In the United States, malnutrition imparts >4 times high odds of hospitalization and an average of US $21,892 more in total charges per stay [3]. It is clear that nutritional status has multidomain effects with both fiscal and clinical ramifications and should be monitored. Older adults (aged ≥65 years) living in long-term care (LTC) homes are especially nutritionally vulnerable, in part owing to low food intake [4]. More specifically, in Canada, 54% of LTC residents are either malnourished or at risk for malnutrition [5]. This is higher than global estimates, ranging from 19% to 42% (37 studies; 17 countries) [6]. Additional independent risk factors for malnutrition are eating challenges and increased cognitive impairment [4,7], which describes between 47% to 90% of the Ontario LTC population [8,9]. Thus, tracking and preventing poor food intake is essential for supporting healthy aging.

However, there is a lack of objective and quantitative tracking methods for food and fluid intake, especially for centralized intake tracking by proxy (ie, multiple staff tracking a set of residents’ intakes). Registered dietitian (RD) referrals are triggered and nutritional support system effectiveness is monitored based on nutritional assessment best practices including unintentional weight loss and usual low intake of food [10]. Resident food and fluid intake charting completed by either personal support workers or nursing assistants captures intake across a meal via visual assessment within 25% incremental proportions at the end of the meal, but may be completed hours later owing to multiple competing priorities during mealtime. Therefore, owing to inconsistency and subjectivity in charting methods, approximately half of residents who would benefit from an intervention are missed [11,12].

Furthermore, there is a lack of trust in current methods because they are known to have poor accuracy and validity [13,14], thus limiting clinical utility. However, it raises awareness to some extent, regardless of whether the measurements are inaccurate (eg, food spills). Measuring food intake is a proxy for nutritional status; however, it provides a sense of *why* something may be going wrong (in combination with biomarkers). Better, more reliable measurements will enable more meaningful assessment of probing when, how, and why something may be going wrong to better inform intervention strategies, and care providers have expressed a desire to leverage high-quality data, provided they are reliable and trustworthy [15].

### Objectives

Automated tools may provide a palatable solution that removes subjectivity and has higher accuracy than human assessors. This may also enable time-efficient measurement of food intake at the energy, macronutrient, and micronutrient levels [15]. More specifically, in LTC, it is desirable to have a high level of detail including intake breakdown for each item consumed (not averaged across a plate) [15]. To estimate food intake and nutrient consumption, 4 main questions must be answered: *where* is there food on a plate (segmentation), *which* foods are present (classification), *how much* food was consumed (preprandial and postprandial volume estimation), and *what* was the estimated food and nutrient intake? This study builds on previous studies exploring *where* food is and *how much* food was consumed at a bulk intake level by leveraging a specialized food segmentation method powered by deep learning for automated segmentation, moving from bulk food segmentation to nutritional estimation with a few additional steps modularized for systematic error assessment [16]. Here, we focused on *which* foods are present and *how much* food was consumed for enabling assessment of *what* was the estimated food intake at the nutrient level.

The purpose of this study was to describe the final stage of feasibility testing of the automated food imaging and nutrient intake tracking (AFINI-T) system comprising pixel-wise food classification and nutrient linking through intake prediction, for providing food and nutrient intake estimation with specific feasibility considerations for use in LTC. Our proposed AFINI-T technology measures food intake compared against gold-standard ground truth weighed food records, addresses automatic segmentation with integrated red-green-blue-depth (RGB-D) assessments, was evaluated in both regular texture foods (RTFs) and modified texture foods (MTFs), and describes the valence of the system within the user context.

## Methods

This study used an iterative action research design, blending mixed methods needs assessment with technical implementation and experimental evaluation.

### Ethics Approval

This study received ethics clearance from the University of Waterloo’s Office of Research Ethics Board (23124).

### End User Data to Shape Technological Requirements—A Case Study

Insights motivating the technical approach described in this paper were gathered through interviews and workshop discussions with Schlegel Village team members during our previous user study, but not included in the paper [15]. Overall, 2 interviews (an RD nutrition research expert and an RD working in LTC) and discussion with experts during a workshop were conducted. The workshop included 21 participants representing 12 LTC and retirement homes who were recruited through self-enrollment, including an administrative assistant, chef, dining lead (similar to a dining room manager), director of recreation, dietary aides, neighborhood coordinator, recreation assistant, restorative care, senior nurse consultant, directors and assistant directors of food services, registered nurse, and personal support workers [15]. Participants identified potential barriers to uptake including time and whether the level of detail is desired or seen as valuable. Qualitative results from interviews and workshops with end users illuminated the following user needs, which, guided by grounded theory [17], were translated into design requirements for application within the LTC context.

### Experimental Procedure—AFINI-T’s Technical Approach

#### Data Collection

**Figure 1:**
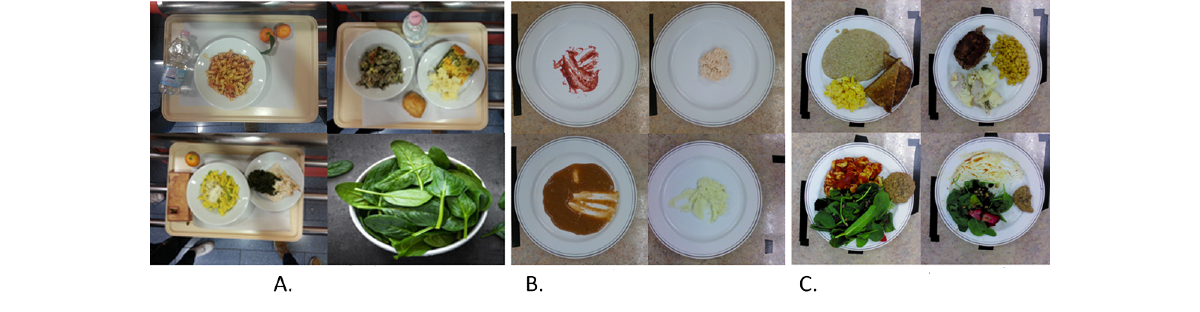
Example images in the data sets used for training the convolutional autoencoder (ie, UNIMIB+ [UNIMIB2016 with additional green representation]) [18,19] and food classification training and testing on modified and regular texture foods. A: UNIMIB+; B: Modified texture foods; C: Regular texture foods.

As described in the study by Pfisterer et al [16], data were collected in an industrial research kitchen at the Schlegel University of the Waterloo Research Institute for Aging’s Centre of Excellence for Innovation in Aging. This kitchen was modeled after industrial research kitchens found in LTC homes. RGB-D images were acquired using Intel RealSense (F200), with a depth resolution of 640×480 pixels. A sequence of 10 depth images was acquired for each plate and averaged to reduce pixel noise. An optical imaging cage was constructed to enable top-down image capture, as described in the study by Pfisterer et al [16]. The camera was connected to a computer for data acquisition, and plates were weighed at a nearby weigh station. Figure 1 shows examples of the data sets used for training the convolutional autoencoder and food classification network, which are described in detail in the following subsections.

#### RTF and MTF Data Sets

We used our RTF data set (9 foods; 9 classes; 375 images) and our MTF data set (47 foods; 93 classes; 664 images). Table 1 provides an overview of data set characteristics, and a summary of all food items imaged can be found in Table 2. Our RTF data set comprised 3 *meal* plates, each consisting of 3 foods imaged at every permutation of 25% simulated intake. Our MTF data set consisted of 134 food samples representing 47 foods, each consisting of a set of at least one purée and one minced texture food. Each sample was imaged 5 times by progressively removing food, with the exception of 4.5% (6/134) of the samples consisting of 4 each with 1 lost image.

For each food item, 1 full serving was defined by the nutritional label portion size (RTF data set) or the recipe-defined portion size received from the kitchen and was weighed to the nearest 1 g using an Ohaus Valor Scale.

For the RTF data set, in which a serving size was referenced using volume, that volume of food (eg, corn) was weighed, and the mass was used thereafter. As manufacturers supply nutritional information for minerals as percentage of daily value (assuming a 2000-calorie diet), for the RTF data set, minerals were reported similarly. For more details on conversion, refer to Table S1 in Multimedia Appendix 1 [20]. Mass in grams was used to define all serving sizes.

For the MTF data set, we expanded our original MTF data set [16] with additional examples (that did not include recipes) for further segmentation and volume estimation analysis. Nutritional analysis was conducted on a subset of 47.3% (314/664) of the images. As nutritional information was provided according to mass, we converted from mass to volume. To accomplish this, we calculated the food’s density to convert by using the full plate’s *true volume* (in mL) with its mass (in grams). This enabled the scaling of nutritional information using the RTF data set pipeline for validating these findings using mass; it was not required for the system to operate.

**Table 1:** Overview of data set characteristics. The UNIMIB+^a^ data set was used for training and validation [18,19].

Data set overview	UNIMIB+	RTF^b^	MTF^c^	RTF+MTF
Number of images	1214	375	664	1039
Number of samples	N/A^d^	3	134	137
Number of classes	76	9	93	102
Number of foods represented	76	9	47	56
Number of foods with recipes	N/A	9	27	36

^a^UNIMIB+: UNIMIB2016 with additional green representation.

^b^RTF: regular texture food.

^c^MTF: modified texture food.

^d^N/A: not applicable.

**Table 2:** List of foods in the RTF^a^ and MTF^b^ data sets used for testing the AFINI-T^c^ system.

Food component	RTF with recipes	MTF with recipes	Additional MTF with segmentations
**Grains**			
	Cheese tortellini with tomato sauce	Bow-tie pasta with carbonara sauce	Basmati rice
	Oatmeal	Macaroni salad	—^d^
	Whole wheat toast	Vegetable rotini	—
**Vegetables and fruits**			
	Corn	Asian vegetables	Beet and onion salad
	Mashed potatoes	Baked polenta with garlic	Cantaloupe chunks
	Mixed greens salad	California vegetables	Green beans with pimento
	—	Greek salad	Grilled vegetable salad
	—	Mango and pineapple	Roasted cauliflower
	—	Red potato salad	—
	—	Sauteed spinach and kale	—
	—	Seasoned green peas	—
	—	Stewed rhubarb and berries	—
	—	Strawberries and bananas	—
	—	Sweet and sour cabbage	—
**Proteins**			
	Meat loaf	Baked basa	Bean and sausage strata
	Scrambled egg	Braised beef liver and onions	Grilled lemon and garlic chicken
	—	Braised lamb shanks	Pork tourtiere
	—	Hot dog wiener	Roast beef with miracle whip
	—	Orange ginger chicken	—
	—	Salisbury steak and gravy	—
	—	Teriyaki meatballs	—
	—	Tuna salad	—
**Mixed dishes**			
	Oatmeal cookie	Barley beef soup	Black bean soup
	—	Blueberry coffee crumble cake	Broken glass parfait (mixed gelatin)
	—	Eggplant parmigiana	Butternut squash soup
	—	English trifle	Cranberry spice oatmeal cookie
	—	Lemon chicken orzo soup	Lemon meringue pie
	—	—	Peach jello
	—	—	Pear crumble cake
	—	—	Roast beef with miracle whip on whole wheat
	—	—	Turkey burger on wheat bun

^a^RTF: regular texture food.

^b^MTF: modified texture food.

^c^AFINI-T: automated food imaging and nutrient intake tracking.

^d^There were varying numbers of items in the data sets.

#### Training Data Set

**Figure 2:**
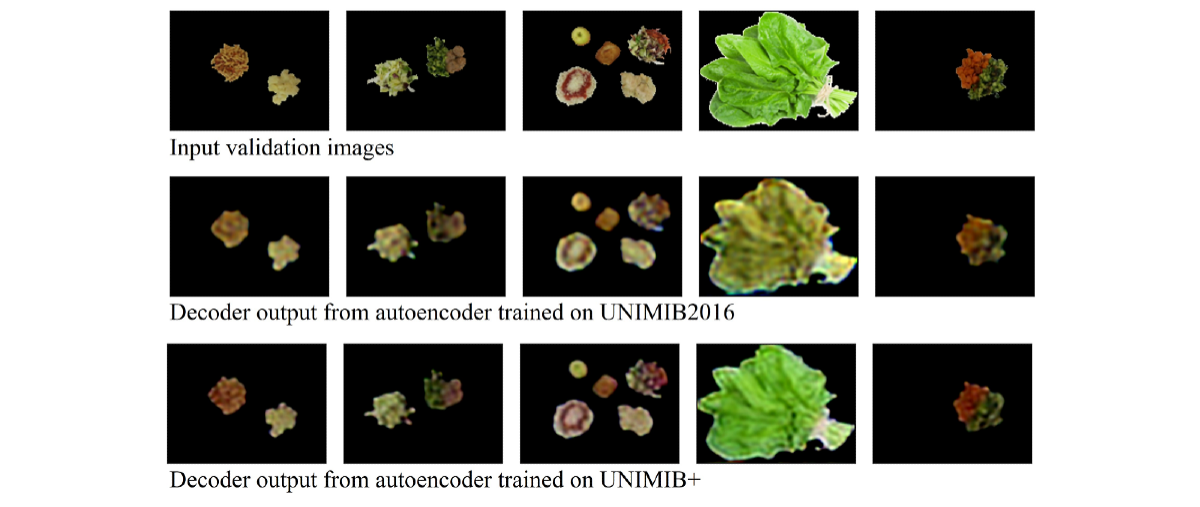
Effect of underrepresentation of green foods in the UNIMIB2016 database on decoder output on segmented food from plates. The decoder output from the autoencoder trained on the UNIMIB+ (UNIMIB2016 with additional green representation) data set in the bottom appears less murky and more vibrant, with truer perceived greens than the UNIMIB2016 counterpart in the middle.

We expanded the UNIMIB2016 data set (1027 tray images; 73 classes) [19] with additional examples from the FoodX-251 data set [18] to train the convolutional autoencoder (described in detail in the *Automation With a Convolution Autoencoder* section). We discovered that UNIMIB2016 had an underrepresentation of green foods compared with what is served in LTC, which affected the autoencoder’s ability to differentiate among all colors and textures. To address this difference in the canteen images from the original UNIMIB2016, we augmented the training data set by adding 91 examples of lettuce, 91 examples of peas, and 89 examples of spinach from the FoodX-251 food data set [18]. Plates with plastic packaging (84/1027, 8.17%) were removed, as they confounded food feature learning and were not representative of LTC plates. We refer to this as the UNIMIB+ (UNIMIB2016 with additional green representation) data set (1214 images; 76 classes). Figure 2 shows the effect of this underrepresentation of green by its inability to reconstruct a vibrant hue across the autoencoder’s decoder output trained solely on the UNIMIB2016 data set for validation examples. The autoencoder was able to converge to low validation loss on the UNIMIB+ data set. Empirically, this resulted in greens appearing greener, reds appearing redder, and yellows and whites appearing less murky, as shown in the UNIMIB+ examples compared with the UNIMIB2016 in Figure 2. This suggests that the addition of the green samples enabled the autoencoder to learn good food representations; encode features more deeply; and align more closely with how a human would perceive the foods, which is a crucial point for the LTC application.

### Computational Methods

**Figure 3:**
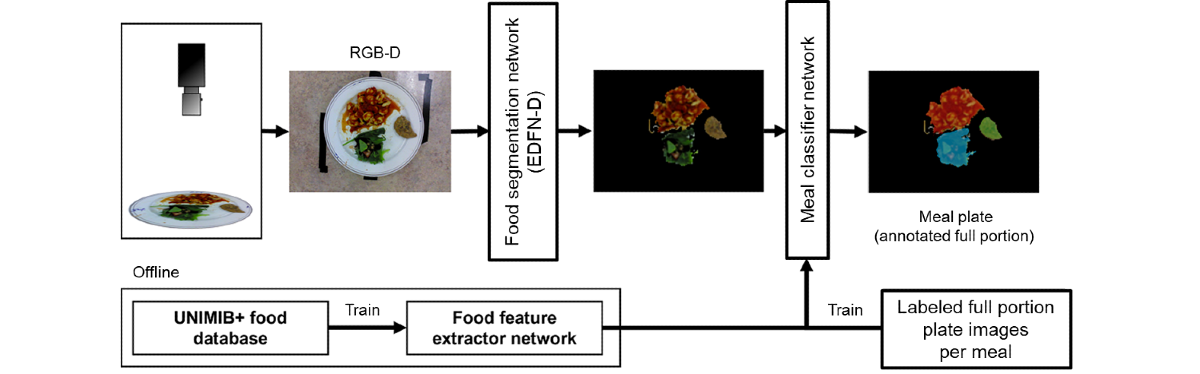
System diagram showing the processing pipeline from image acquisition to food classification. EDFN-D: depth-refined encoder-decoder food network; RGB-D: red-green-blue-depth; UNIMIB+: UNIMIB2016 with additional green representation.

The following sections describe how the segmentation strategy was refined compared with our initial work [16], the general food or no food classification approach, followed by system automation using a convolutional autoencoder. Figure 3 shows the processing pipeline from image acquisition to classified food pixels.

#### Refined Segmentation Strategy

Modifications to the training process were made to enhance network performance. We introduced early stopping criteria to halt training early to avoid overfitting, yielding a network that was trained over fewer epochs than one that is overtrained and outputting a pixel-level image mask as food or no food with calibrated depth [16]. Volume consumed was mapped onto nutritional information for intake approximation. These nutrient-level intake estimates were validated against the ground truth nutritional information obtained through the weighed food method.

#### General Classification Approach

**Figure 4:**
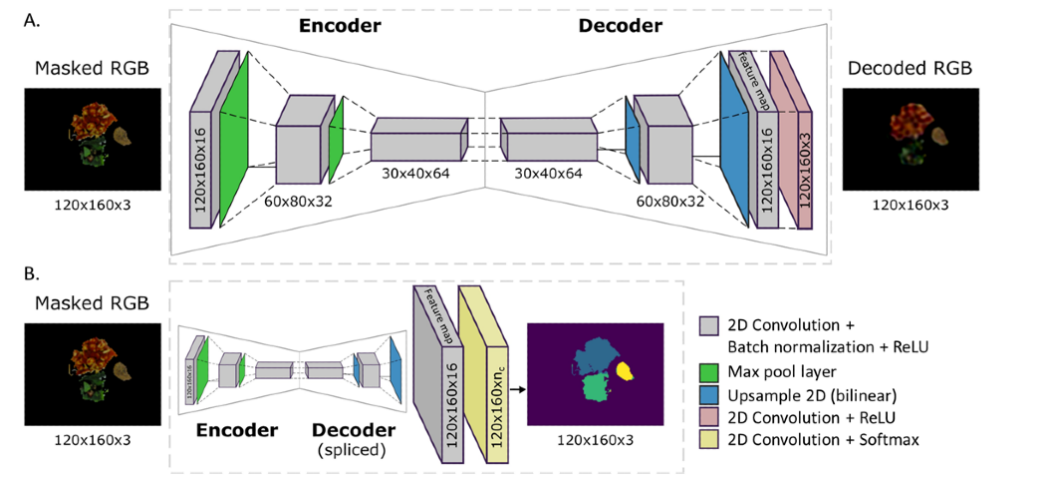
Convolutional autoencoder network for learned feature representation and in the context of classification. (A) The architecture for learning feature representation: an input image is given and the output is a reconstruction of that image. Training minimized the error between input and output images; we used mean squared error loss with Adam optimizer, learning rate of 0.0001, and batch size of 32. The early stop criteria used were change of loss of <0.0001 and patience of 5 epochs. (B) The autoencoder was spliced; weights were frozen; and only a classification layer for n_c_ classes was trained for classification, where n_c_ is the number of food items for meal c. We used categorical cross-entropy (ignoring background pixels) loss, with Adam optimizer and learning rate of 0.1. The early stop criteria used were a change of loss of <1×10^−5^ and patience of 5 epochs. We used 70%:30% train to validation split of augmented data. The data were augmented by generating 300 images from the full set of plates and applying random flips, rotations, and increased or decreased contrast. The outputs are distinct classes, which were mapped onto the meal-specific classifier (in this example, as ravioli [blue], salad [green], and oatmeal cookie [yellow]). ReLU: rectified linear unit; RGB: red-green-blue.

Here, the UNIMIB+ data were used to train the autoencoder. Using the autoencoder’s trained weights, the last layer of the autoencoder (120×160×3) was spliced to use the feature map as a latent feature extractor for classification (refer to Figures 2 and 4 for system diagram and network architecture). This approach was modeled based on our previous study on classification for predicting relative nutritional density of a dilution series of commercially prepared purées [21], because MTF comprises 63.91% (664/1039) of our testing data set and 47% of the LTC population receives MTF [22].

#### Automation With a Convolutional Autoencoder

We report nutrient intake accuracy using the automated system (ie, the automated classification case) to enhance pragmatic feasibility (ie, reduced user input). For this automated approach, we developed a semantic segmentation network with a convolutional autoencoder feature extractor for classification of foods, which was roughly inspired by a highly successful convolutional neural network (CNN), the Visual Geometry Group network [23], in Tensorflow 2.3.0. For a given meal or time of day, we fed the *masked* output from the depth-refined encoder-decoder food network (EDFN; food or no food detector, as described in the study by Pfisterer et al [16]) into the convolutional autoencoder. CNNs encode spatial information, and given how food has differing degrees of cohesion, we felt that the context of spatial information will be an asset. In addition, we sought to extract latent features via a method requiring a round of training offline. For classification, a small classification layer was appended and trained for each meal using a priori information about the meal items offered. Loss for the autoencoder network was computed as pixel-wise mean squared error between the input and reconstructed output; therefore, they did not require labeled training data.

We trained an autoencoder to be a feature extractor using the UNIMIB+ data set consisting of 1214 images. Data were divided into 70% training and 30% validation. Training was performed using the Adam optimizer with batch size of 32, mean squared error loss, and early stopping (<0.0001 validation loss change) with 5-epoch patience. Only food pixels were used in the loss calculation using the ground truth masks. After training, the convolutional autoencoder network was spliced before the final 1×1 convolution block to produce original resolution 16-channel latent feature vectors. The weights of this network were frozen and used as a feature extractor for the classification training.

Given that there are many food options and as new meals are planned, we needed a flexible modular approach, which also enables us to use only 1 labeled example per item; the AFINI-T method uses only 1 full reference portion to classify foods and infer intake. For nutritional intake estimation, we leveraged the homes’ known nutritional information from menu planning software (or supplied by the manufacturer) to link proportional nutrient intake. We assumed that recipes were followed exactly.

Denoting the number of menu items for meal *m* as *n_c_*, the classification network for meal *c* was built by appending *n_c_* 1×1 convolution kernels onto the feature extractor network. The meal full portion training data were constructed by augmenting the full set of plates by applying random flips, rotations, and increased or decreased contrast, yielding 300 augmented instances of the meal. We used 1 reference image (the full portion image) to learn what each class looked like and then mapped subsequent instances onto these prelabeled classes by grouping all the *full plates* of food for a given meal into the training set. The data were divided into 70% training and 30% validation. Training was performed using the Adam optimizer with batch size of 32, categorical cross-entropy loss, and early stopping (<1×10^−5^ validation loss change) with 5-epoch patience. Only food pixels were used in the loss calculation using the ground truth masks. Finally, we applied ground truth labels to the full portion plate to link the proper proportional intake at the nutrient level and assess the accuracy of the intake estimates compared with gold-standard weighed food approach.

### Nutrient Intake Association

This step comprised three general stages: (1) determine the relative consumption of each food item compared with a full reference portion, using food volume estimation from the depth maps; (2) compare relative consumption with nutritional information, to infer nutritional intake for each item; and (3) sum the inferred nutritional intake for each item across a plate for estimation of total nutrition consumed during a meal (for MTF, this was across the plate of one food item).

### Statistical Analyses

#### System Accuracy

Segmentation accuracy was assessed using intersection over union (IOU). Classification accuracy was described using top-1 accuracy and summarized using per-meal classifiers. Bulk intake accuracy (ie, class-agnostic, overall food volume intake) was assessed using mean absolute error (mL) and 3D, % intake error, described in the study by Pfisterer et al [16] in which intake error was calculated for volume (3D) data relative to the full portion. All values are reported as mean (SD). Nutrient intake accuracy was assessed using the fully automated classification approach (ie, without updating misclassified regions) to evaluate nutrition intake accuracy and is reported as mean (SD) and percentage error.

#### Validating Nutrient Intake Estimation Against Weighed Food Records

All data were analyzed using MATLAB 2020b (MathWorks). Linear regression was used to determine the goodness of fit through the degree of correlation with *r*^2^ to summarize the extent to which nutritional intake information from weighed food mass is related to estimated nutritional information from food volume. Bland-Altman analysis was used to describe the level of agreement between nutritional intake information from weighed food mass compared with intake volume using mean agreement (σ) and bias (µ) between methods [24].

Several nutrients of concern in the RTF data set were reported in percentage daily value (ie, calcium, iron, vitamin B6, vitamin C, and zinc). We converted these values to absolute values to match the MTF data set using the 2005 Health Canada reference values for elements and vitamins. Where there was a difference across age, we used the reference for age >70 years; where there was a difference in requirement by sex, we used the average value.

## Results

### Overview

This study focused on the characterization of changes in volume at the whole plate level for bulk intake estimation, reporting degree of consumption (ie, proportion of food consumed) and nutritional intake estimation using a nutritional lookup table at the food item and whole plate level. Specific needs informed by workshop and interview responses included the following:

1. The system shall consider evidence-based and practice-relevant priority nutrients (output: 13 nutrients of interest—macronutrients: calories, carbohydrates, fats, fiber, and protein and micronutrients: calcium, iron, sodium, vitamin B6, vitamin C, vitamin D, vitamin K, and zinc).
2. The system shall support current workflow in which the dietitian is the gatekeeper:
  The system shall facilitate automated, objective, intake estimates.
  The system shall facilitate dietitian referrals by providing repeatable nutrient-specific intake insights.
3. The system shall work independently of internet connection.
4. The system shall incorporate real-world constraints and parameters:
  The system shall include a salient feature extractor that can be trained in advance and supports real-time use.
  The system shall use a classification method that is light in weight for mobile app use.
  The system shall include an easily updatable classifier to account for a priori menu plans considering the time of day and therapeutic diet.

The following quantitative results provide an overview of the AFINI-T system’s food and nutrition intake estimation system including segmentation, classification, volume estimation, bulk intake, and nutrient intake accuracies.

### Segmentation Accuracy

Table 3 provides an overview of segmentation accuracy. Generally, results represent 2 types of meal scenarios: multiple RTF data set on a plate and single MTF data set on a plate. The RTF data set had 9 unique foods across 375 simulated intake plates. The MTF data set foods were prepared by the LTC kitchen and included 93 unique foods including both purées and minced foods across 664 simulated intake plates. Across the RTF and MTF data sets, there are 102 classes represented in 1039 simulated intake plate images. Segmentation accuracy was good with an average IOU of 0.879 across the RTF and MTF data sets (Table 3). Segmentation accuracy ranged from 0.823 for the MTF data set at lunch to 0.944 for the RTF data set for breakfast. From the perspective of IOU, the MTF data set was more poorly segmented by the depth-refined EDFN; however, consistent with the study by Pfisterer et al [16], the degree of visual-volume discordance was high for modified texture diets and is discussed further in the Volume Estimation Accuracy section.

**Table 3:** Average segmentation and classification accuracies within and across data sets^a^.

Data set and meal		Classes (N=102), n	Images (N=1039), n	Segmentation accuracy (IOU^b^), mean (SD)	Classification accuracy (top 1), %
**RTF^c^**		9	375	0.929 (0.027)	93.9
	Breakfast	3	125	0.944 (0.019)	93.5
	Lunch	3	125	0.919 (0.033)	93.5
	Dinner	3	125	0.928 (0.019)	95.1
**MTF^d^**		93	664	0.879 (0.101)	88.9
	Day 1—lunch	5	25	0.841 (0.123)	89
	Day 1—dinner	15	90	0.823 (0.099)	70.2
	Day 2—lunch	12	74	0.863 (0.118)	70.6
	Day 2—dinner	12	90	0.840 (0.122)	64.9
	Day 3—lunch	10	85	0.834 (0.132)	80.4
	Day 3—dinner	15	109	0.859 (0.100)	70.4
	Day 4—lunch	9	60	0.871 (0.113)	72.2
	Day 4—dinner	10	90	0.837 (0.107)	67.8
	Day 5—lunch	5	41	0.881 (0.117)	87.8

^a^There were no samples for day 5–dinner.

^b^IOU: intersection over union.

^c^RTF: regular texture food.

^d^MTF: modified texture food.

### Classification Accuracy

As shown in Table 3, classification accuracy was high for the RTF data set, with top-1 accuracy (ie, the most likely class) ranging from 93.5% for breakfast and lunch to 95.1% for dinner. However, the RTF data set had only 3 classes per meal; therefore, it was a less challenging classification problem compared with a great number of classes to differentiate among, especially when considering the MTF data set had less texture variance. In contrast, the MTF data set top-1 accuracy ranged from 64.9% on day 2–dinner with 12 classes to 89% on day 1–lunch with 15 classes.

### Volume Estimation Accuracy

**Figure 5:**
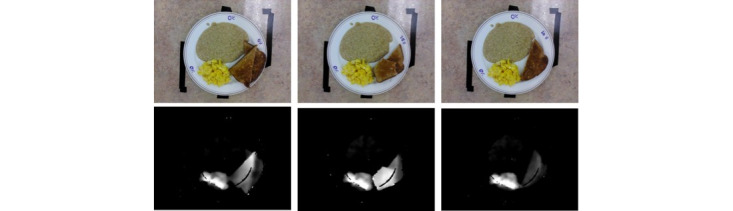
The occlusion conundrum, as demonstrated by stacked toast with an overhang. As volumetric food estimation is based on pixel-wise classification, the pixels of the overhang are assumed to contain toast. This is a limitation to overhead imaging and provides a simplified example of low-density foods (eg, salad) as does rigid toast placed as an inclined plane. This is seen in the depth images; bright parts denote pixels close to the camera (ie, high food pixels). We see a gradient from low to high near the tip with a similar, but less obvious, trend in the third depth image. The depth map range was adjusted to exemplify the toast height.

Low-density foods pose challenges to depth scanning systems. Here, volume estimation was within tolerance with food volume error of 2.5 (SD 9.2) mL, and low-density foods (eg, salad) have the largest food volume error seen for RTF: lunch of −10.1 (SD 22.2) mL. A similar issue of low-density foods is seen through the 3D, % absolute error intake of 14.4% (SD 13.1%), which we suspect is owing to the air pocket below some of the pieces of toast that are placed at a tangential angle to the plate or when 2 pieces are stacked with overhang, as shown in Figure 5. This can be considered as one of the classic examples of the *occlusion conundrum* with the imaging limitation of collection from an overhead view. This is an example of where segmentation can be performed perfectly, but will translate to volume estimation errors.

### Bulk Intake Accuracy

Table 4 summarizes the bulk intake accuracy within and across data sets. Compared with the study by Pfisterer et al [16], for this iteration, we incorporated more representation of green in the UNIMIB+ data set for training and validation and introduced a more optimal stop criteria for training for segmentation. In the study by Pfisterer et al [16], we saw that the mean absolute volume error was 18 (SD 50) mL for RTF and 2.3 (SD 3.2) mL for MTF and mean volume intake error was 130.2 (SD 154.8) mL and 0.8 (SD 3.6) mL for RTFs and MTFs, respectively. Here, accuracy is higher with mean absolute food volume error of 6.6 (SD 13.6) mL for RTFs and 2.1 (SD 3.1) mL for MTFs. Similarly, the bulk intake accuracy was higher, with mean absolute intake error greatly reduced for the RTFs (39.9, SD 39.9 mL), but slightly higher for MTFs (6, SD 5.6 mL). The higher degree of visual-volume discordance for MTFs compared with RTFs is again corroborated in Table 3, with mean food volume error of 3.8 (SD 8.8) mL and higher mean volume error for the RTF data set (6.6, SD 13.6 mL) than for the MTF data set (2.1, SD 3.1 mL).

**Table 4:** Bulk intake accuracy within and across data sets^a^.

Data set and meal			Classes (N=102), n		Images (N=1039), n		Food volume error				Bulk intake accuracy				
							Absolute error (food volume; mL), mean (SD)		Absolute error (intake; mL), mean (SD)		Error (intake; mL), mean (SD)		3D, % absolute intake error, mean (SD)		3D, % intake error, mean (SD)
**RTF^b^**			9		375		6.6 (13.6)		39.9 (39.9)		–7.2 (56)		13.1 (10.9)		–2.5 (16.8)
	Breakfast	3		125		3 (4.1)		17 (14.3)		−15.1 (16.3)		14.4 (13.1)		−12 (15.3)	
	Lunch	3		125		11 (21.7)		76.1 (48.5)		18.1 (88.7)		13.7 (9)		7.6 (14.6)	
	Dinner	3		125		6 (6)		26.5 (14.4)		−24.5 (17.7)		11.2 (9.9)		−2.9 (14.7)	
**MTF^c^**			93		664		2.1 (3.1)		6 (5.6)		4.4 (6.9)		7.6 (8)		5.9 (9.4)
	Day 1—lunch	5		25		1 (1.1)		3.4 (3.3)		−0.9 (4.7)		5 (4.6)		−0.3 (6.9)	
	Day 1—dinner	15		90		1.9 (2.9)		4.1 (3.7)		2.5 (5)		7.4 (14.1)		6.3 (14.6)	
	Day 2—lunch	12		74		2.2 (3.3)		7.4 (7.3)		6.1 (8.4)		6.7 (5.5)		5.1 (7)	
	Day 2—dinner	12		90		1.2 (1)		4.6 (4.3)		2.9 (5.5)		8.3 (7.7)		5.5 (9.9)	
	Day 3—lunch	10		85		3.8 (5.1)		7.6 (6.3)		5 (8.5)		11.5 (10)		10 (11.5)	
	Day 3—dinner	15		109		1.9 (2)		5.5 (3.8)		3.9 (5.4)		6.7 (4.7)		4.9 (6.6)	
	Day 4—lunch	9		60		1.5 (2.5)		5.6 (7.5)		4.8 (8)		6.3 (3.9)		5.3 (5.2)	
	Day 4—dinner	10		90		2.1 (1.9)		6.5 (4.8)		5.8 (5.6)		6 (4.7)		5 (5.8)	
	Day 5—lunch	5		41		3.4 (5.2)		9.5 (6.7)		7.8 (8.6)		9.9 (5.9)		7.7 (8.7)	
RTF+MTF			102		1039		3.8 (8.8)		19.9 (30.8)		–0.4 (36.7)		9.9 (9.7)		2.4 (13.6)

^a^There were no samples for day 5–dinner; *food volume* error is equivalent to *mean error bias*; *error (intake)* is equivalent to *volume intake error*; and *3D, % intake error* is the same as in the study by Pfisterer et al [16]

^b^RTF: regular texture food.

^c^MTF: modified texture food.

### Validating Nutrient Intake From Volume With Mass

**Figure 6:**
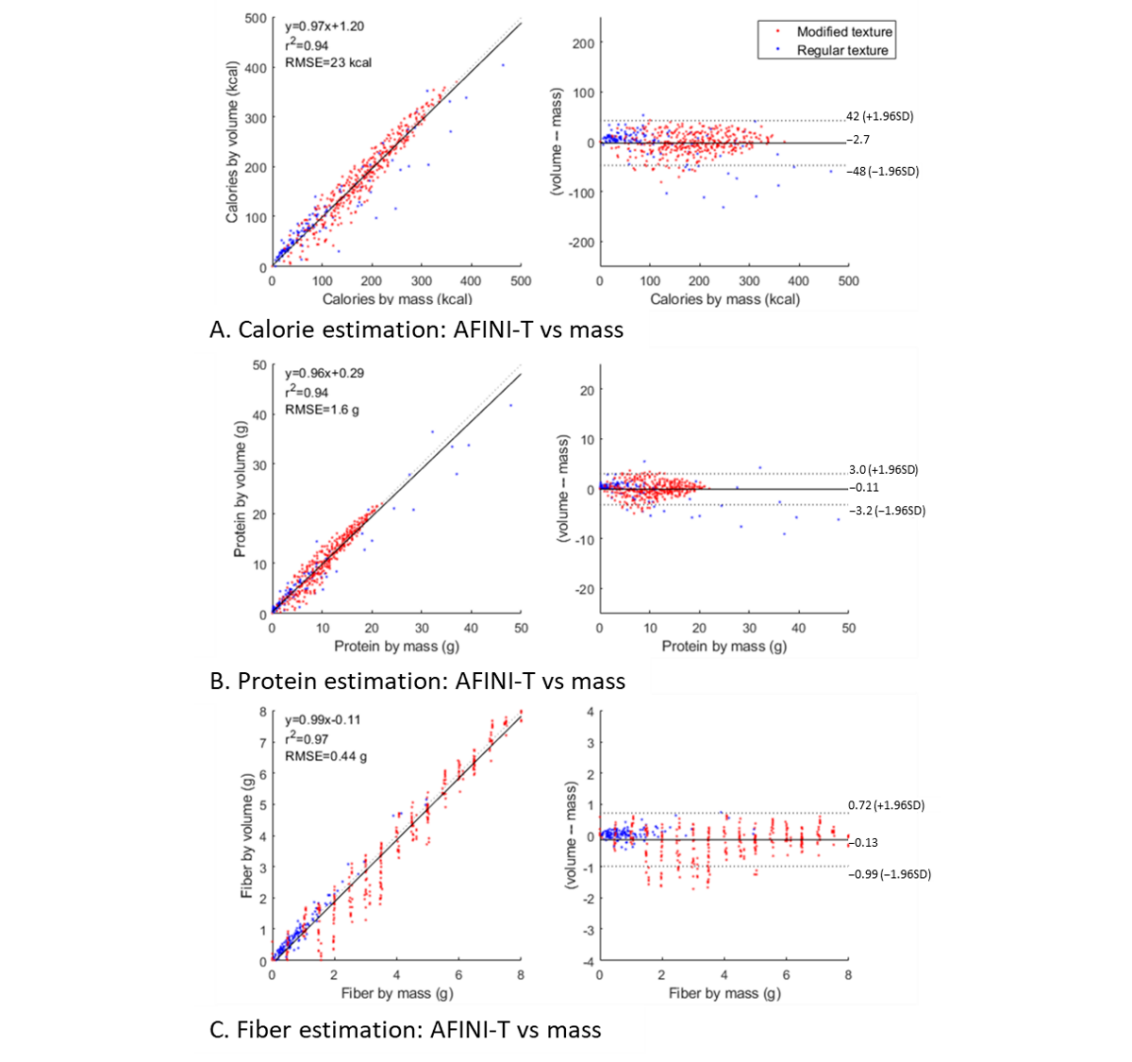
Correlation and agreement between mass and volume estimates for determining nutritional intake at the whole plate level across all imaged samples. Left panel depicts the goodness of fit with linear regression and coefficient of determination (r^2^), and right panel depicts the degree of agreement between measures and bias from the Bland-Altman method. Correlation and agreement between mass and volume estimates of macronutrients are shown in the figure: (A) calories, (B) protein, and (C) fiber. In total, 3 nutrients of interest are shown here for brevity. RMSE: root mean square error.

In Figure 6, the MTF plates (blue) tended to be of lesser mass than the RTF plates (red), largely owing to the nature of RTF. RTFs represent available food choices from the LTC home, but they were prepared by a supermarket, which may not be consistent with LTC serving sizes. MTF were offered and prepared by the LTC home. This translates to a clustering effect of MTF foods at lower values of nutrients with RTF foods toward higher values of nutrients. We also observed a banding effect on fiber for the RTF data set owing to how mass was controlled for matching 25% portion increments and given the relatively few foods that contained fiber in the RTF data set. Regarding the spread of nutrient distributions, there is also much higher variance for the MTF data set for larger amounts of a nutrient (eg, protein, fat, and iron), with tighter variances observed on smaller portion sizes.

On the basis of the coefficients of determination shown in Figure 6, nutrient estimates by volume were tightly linearly correlated with nutrient estimates from mass, with *r*^2^ values ranging from 0.92 for fat to 0.99 for vitamin C and vitamin K. This was true for all nutrients of interest (refer to Tables S2-S4 and Figure S1 in Multimedia Appendix 1) for a comprehensive assessment). On the basis of the Bland-Altman plots, not only were they tightly correlated but there was also good agreement between methods, as evidenced by small bias (|µ|≤2.7) and 0 contained within the limits of agreement. Ideally, the bias distributions will be centered around the y-intercept (ie, µ=0). This was the case with µ ranging from a minimum of −0.01 for vitamin B6 (mg), zinc (mg), and fat (g) to a maximum of −2.7 for calories (kcal). Taken together, these results suggest that nutrient estimation using the AFINI-T system appears to be valid. Estimates were well aligned with the gold-standard weighed food method, with the advantage of only single image acquisition and no need for weighing plates.

### Benchmarking the AFINI-T Approach With Current Practice and Requirements

Now, let us consider the feasibility of theoretical portability and completion task time by comparing the end-to-end AFINI-T system with the current workflow. A requirement identified in the study by Pfisterer et al [15] was for the system to run on a portable tablet with inconsistent Wi-Fi. By design, methodology and models were selected to support portability. For example, having selected an approach to support offline training in the EDFN and autoencoder, only the final model residing on the device, which does not require Wi-Fi. The autoencoder, which requires only a single training session for global feature extraction, encompasses 84,176 parameters. The per-meal classification layer requires an additional 15*n_c_* trainable features, where *n_c_* is the number of food classes for meal *c* (Table 3). The EDFN food detection network requires 13.7 million parameters, but does not require fine tuning and can be used globally across meals.

The second benchmark is regarding theoretical task completion time. In terms of benchmarking theoretical task completion time, we can compare with results from the study by Pfisterer et al [15]. When assuming a very conservative estimate including food handling of 10 seconds per image for acquisition, the time for preprocessing (eg, plate finding) takes approximately 2.5 seconds per image, with segmentation taking 0.7 seconds per image and classification taking 0.05 seconds per image (Dell XPS 15 9570; i7-8750H 2.20 GHz 6-core central processing unit; Nvidia GeForce GTX 1050 Ti). As shown in Table 5, even based on these conservative estimates, the theoretical completion time using AFINI-T meets the low end of task completion times (9 minutes 45 seconds vs a mode of 10-14 minutes of completion time for charting 1 meal). Here, we have assumed separate imaging for each of the appetizers, mains, and desserts for each resident. Instead, if we consider acquisition as only acquiring the image (estimated time 1 second), this drops to 2 minutes 34 seconds. The true completion time will likely be between these upper and lower bounds, but the key point is that AFINI-T is platformed to take less time than the current methodology and with the added benefit of being objective and capturing data at a resident-centric level. Instead of a resident’s intake being binned into the 25% bin across the average foods served that day, AFINI-T captures details at the mL level and tracks personalized items ordered on a resident-by-resident basis.

**Table 5:** Summary of length of time required to complete food and fluid intake charting for 1 neighborhood (unit) comprising 16 residents, compared with theoretical AFINI-T^a^ processing.

Type	Mode time (minutes)	Responses, n (%)	Time range (minutes)	AFINI-T estimate (1-second acquisition)	AFINI-T estimate (10-second acquisition)
Food (per meal)	10 to 14	3 (33)^b^	<10 to >25	2 minutes 34 seconds	9 minutes 45 seconds
Fluid (per meal)	10 to 14	4 (40)^c^	<10 to 25	N/A^d^	N/A
Snack (per snack)	<10	5 (55)^b^	<10 to 19	52 seconds	3 minutes 15 seconds

^a^AFINI-T: automated food imaging and nutrient intake tracking.

^b^Sample size, n=9.

^c^Sample size, n=10.

^d^N/A: not applicable.

## Discussion

### Principal Findings

The AFINI-T method for estimating food intake is in strong agreement and tightly correlated with true intake. Especially in the case of larger intake portions, the AFINI-T method yielded accuracy of nutrient content with <5% error. For context, comparison with current visual assessment methods indicate errors in portion size 56% of the time for immediate estimation and as low as 62% for delayed recording and stating that current methods’ error is too high for accurately identifying at-risk residents [25]. Interpretation of the acceptability of the precision and accuracy of the system requires further input from users, ideally through pragmatic trials. If warranted, improvements will require a degree of human input or expanded models. This may be in the form showing output classification masks so that misclassified segments can be reclassified as appropriate. Alternatively, it can be used to seed food item regions to tightly constrain food regions and then apply region growing to *intuit* where there are food segments. This approach is consistent with what was integrated into the collaborative co-design prototype development outlined in the study by Pfisterer et al [15]. Although not fully automatic, collaborative segmentation through machine learning estimation that is checked and corrected, if necessary, by a human using a simple and intuitive interface will likely be an improvement on current food charting methods, particularly regarding accuracy and time. Timed comparison trials will be required to confirm this.

For the current AFINI-T approach, we show that segmentation of only 1 reference image is required and that even when some pixels are misclassified, there is reasonable robustness in nutrient intake accuracy. These misclassifications tended to occur near the edges of a food segment regardless of data set, which may be from a less uniform representation near the edges either because of higher crumbliness (eg, meat loaf crumbs) or owing to the convolutional kernel extending into the *empty space* (ie, the plate), making it easier to classify a pixel as food when there are food pixels surrounding it. These misclassification errors at the edges do not appear to translate to large intake errors. This fully automated classification strategy may be deemed feasibly acceptable given the time savings. It is also consistent with the co-designed user interface and workflow we reported in our previous study, where users described acceptability for clicking on a large food region and defining its contents from a drop-down list [15] which can be prepopulated based on the menu items of the day.

In the case of frequent nutrient database missing values (eg, vitamin D [26]), there is reliance on complex imputations for estimates [27]. Additional discussions with end users and nutrition experts are warranted to evaluate the utility and appropriateness of reporting these values, the margin of error that is deemed acceptable for supporting trust in the system, and other considerations given the quality of data included in the underlying nutritional databases.

### Comparison With Previous Studies

It is challenging to assess how AFINI-T compares with the literature because there are no food *intake* data sets on which benchmark tests can be conducted. Additional considerations affecting the ability to compare include the number of included classes, inconsistencies in *accuracy* reporting (eg, top-1 vs top-4 accuracy), and the complexity of the classification problem (eg, whole raw foods vs prepared meals modified texture versions of those prepared foods). Although direct comparison between the AFINI-T system and other automated methods for assessing LTC intake data is not possible because the AFINI-T system is the first to measure food intake and consider MTFs, these results suggest that AFINI-T’s deep neural network approach is among the highest performing approaches with a top-1 accuracy of 88.9%. Furthermore, the type of data represented in the MTF and RTF data sets for LTC contain more complex food scenarios, as they are prepared foods (RTF: 93.9% accuracy; MTF: 73.7% accuracy), and the accuracy we report is top 1, which means that the AFINI-T approach may outperform the others.

Some accuracy for classification methods based on handcrafted features has been reported in the literature: 85% accuracy for 15 types of produce with minimum distance classifier [28], 88.2% accuracy for 18 classes of whole foods (entire pineapple) [29], 95% using top-4 accuracy for supermarket vegetable identification [30], 96.55% accuracy for 10 vegetables using a neural network with color and texture features [31], and 99% using top-2 accuracy for some fruits and vegetables by fusing 3 types of features (including Unser’s features) [32]. Regarding the trend for learned features, a deep learning approach has had comparatively slow adoption in the field of food imaging, with uptake occurring only recently [33-35].

Regarding accuracy reporting for segmentation and classification, these accuracies tend to not be mentioned [35-39] or are stated as beyond the scope of the present version of their system [37]. This is further confounded when segmentation and classification accuracies are combined instead of considering them as 2 subprocesses. Classification accuracies using deep learning vary from as high as 100% (11 classes) [34] to 82.5% (15 classes) [35]. Alternative methods used for classification were AdaBoost [37], K Nearest Neighbors [40], and support vector machines [36,39], with reported classification accuracies of 68.3% (50 classes) [36] to 99.1% (6 classes) [39]. At the inference level, few papers report percentage error at the nutrient level and tend to focus on calorie estimation or nonstandardized metrics: calorie estimation error of 0.09% (mean absolute error) on 6 categories using random forests and support vector machines [39] and 0.25% (mean SE) on 11 categories of entire foods (eg, green pepper) using a CNN [34]. Others have reported 80% of calorie estimates falling within 40% error (35% within 20% error) on 15 classes using a multitask CNN, with a maximum correlation coefficient of 0.81 (*r*^2^=0.64 equivalent) and top-1 accuracy of 82.48% [35]. Previous study [35] also reports a comparison with the study by Miyazaki et al [37], with 79% of calorie estimates falling within 40% error (35% within 20% error) using handcrafted features, with a correlation coefficient of 0.32 (*r*^2^=0.10 equivalent).

For comparison, the AFINI-T system demonstrated an error of 2.4% across 13 nutrients in 56 categories (102 classes) of food with minimum *r*^2^ value of 0.92 (0.94 for calories). The average top-1 accuracy was 88.9%, ranging from 95.1% for 3 classes (RTF: dinner) to 70.4% and 89% for 15 class meals (MTF: day 3–dinner and MTF: day 1–lunch, respectively). On the basis of these comparisons, this study performs among the best reported in the literature, despite having more complex meal scenarios across 13 nutrients. Although there has been relatively little work done in this area, these results represent a novel contribution both from the technical implementation and real-world implementation perspectives. Additional benefits of the AFINI-T system include its ability to measure a specific resident’s intake (as opposed to the proportion consumed across the average of all foods offered), with performance at least matching other approaches. Compared with the current visual assessment methods, it is easy to use, is fast to acquire and process, removes subjectivity, provides repeatable estimates, and can be tracked to the nutrient level to provide a comprehensive profile of each resident-specific intake in a quantitative way. This translates into high-quality data that can be used to inform resident preferences and streamline referrals to RDs, along with a data-driven approach for monitoring and evaluating nutritional interventions.

### Limitations

First, ground truth volume was assumed to be equivalent to the RGB-D camera assessment. Although we collected ground truth weighed food records, as this study aimed to assess overall feasibility from an accuracy perspective through the lens of end users, we did not account for ground truth volume. Therefore, we were working under the assumption that AFINI-T’s volume assessment was accurate. Volume validation against gold-standard ground truth (eg, water displacement) is needed to corroborate the accuracy (although in actuality, there is some evidence suggesting there is <3% volume error of the RealSense [41]). This is an important consideration for more thoroughly quantifying error at each stage. Given the state of the literature on how error is typically reported (if it is at all), this paper provides evidence of the feasibility of more transparent technology for supporting trust in the system.

Second, although the plated foods are representative of LTC offerings, intake was physically simulated through incremental plating in the research kitchen by the researchers. Further studies need to be conducted to evaluate the imaging technology in real-world LTC resident food intake.

### Future Directions

Future directions include adding an additional stage for automatic food type classification as specific foods rather than arbitrary classes with associated nutritional values (ie, mashed potatoes are classified as mashed potatoes after the initial segmentation step). A human-in-the-loop version, where there is the opportunity to correct all misclassified regions (ie, the best-case scenario), can further improve results, albeit at the expense of manual hands-on time and effort, which needs to be minimized. In addition, improving the algorithms to handle more complex food types (eg, salads or soups in which the food comprises multiple components) and more complex plates of food to address food mixing as seen with mashed potatoes will improve AFINI-T’s ability to assess plates *in the wild*.

As observed in Figure 6, with small intake amounts and therefore small relative portion differences, the error was large. To improve on this, in the future, we must consider from where this error arose. A contributor may be the depth map variance. Results indicate that nutritional intake estimates had great variation at low levels of intake (large spread at low intake levels). This may correspond to the amount of variation in estimation at low levels of intake or large quantities of food left on the plate. We speculate that this is because of compounding of small discrepancies in depth maps, which get propagated to volume and then to nutritional intake. Future studies will address this issue by incorporating depth map variance as a feature to describe the food item. For example, for a green salad, we expect a higher variance in the depth map because it is a nondense food item. In contrast, meat loaf or slab cake will have very low depth map variance across the food item, as these items are more similar to a block. Exploring automated 3D segmentation may also be intriguing, in which depth information can be stacked onto color channels and thus incorporated into salient feature extraction. Similar approaches have shown promise in recent advances in agriculture [42-44], construction [45], robotics, and automation [46].

From a translational standpoint, AFINI-T is platformed to provide actionable data-driven insights that can help to inform menu planning by dietitians and director of food services. For example, it can be used to develop recipes that are more nutrient-dense and complement the nutrients in recent past meals. Creating nutrient-dense meals while minimizing cost is a priority in LTC, as there is a fixed allocation of food cost per resident. The raw food allocation in Ontario was CAD $9.54 (US $6.82) per resident per day in 2020 [47]. Until recently, there was a disconnect between the perceived requirement to serve full portion to meet nutritional requirements (ie, the portion size that was costed to provide adequate nutrition); however, because of limited budget, the foods that were served were relatively inexpensive and the quantity required was unsuitable. This resulted in high degree of food waste [48,49], increasing the risk for malnutrition owing to less consumed nutrients than the planned nutrient consumption [50]. AFINI-T can also be used as a tool for developing more nutrient-dense recipes in which certain ingredients can be replaced with others. For example, replacing half of the ground beef in a chili recipe with lentils to decrease saturated fat and cholesterol and increase fiber. Data on which foods are consumed can inform how to design recipes to be smart, more expensive, and more nutrient-dense, with the expectation of less waste and more portion consumption, especially when paired with software such as Food Processor for designing recipes. Although these types of strategies were not part of this study, they are direct motivation for this project and have great potential to affect and disrupt the way we assess nutrition management and beyond when they are explored as part of future pragmatic trials.

### Conclusions

AFINI-T is a feasible deep learning–powered computational nutrient sensing system that provides an automated, objective, and efficient alternative for food intake tracking, which provides food intake estimates. Novel contributions of this approach include a novel system with decoupled segmentation, classification, and nutrient estimation for monitoring error propagation and a convolutional autoencoder network for classifying regular texture and MTFs with top-1 accuracy of 88.9%, with mean intake error of 0.4 (SD 36.7) mL, and nutritional intake accuracy with strong agreement with gold-standard weighed food method and good agreement between methods (*r*^2^ ranges from 0.92 to 0.99; σ ranges from −2.7 to −0.01; 0 within the limits of agreement) across 13 nutrients of interest to LTC. Translation of AFINI-T may provide a novel means for more accurate and objective tracking of LTC resident food intake, thus providing new resident-specific insights for supporting well-being and preventing malnutrition. AFINI-T’s data-driven insights may streamline and prioritize dietitian referrals for supporting nutritional intervention efficacy. This may enhance the sensitivity of identifying at-risk residents and enable more holistic monitoring for malnutrition reduction.

## Appendix

Multimedia Appendix 1 This multimedia appendix contains two parts: S1—standardizing nutrient values and S2—nutrient intake accuracies. S1 consists of Table S1, describing the workflow in converting percentage daily values to absolute measurements using Health Canada’s dietary reference intake values [20]. S2 contains supplementary tables on nutrient intake accuracies (Tables S2-S4), providing a comprehensive overview of nutrient intake accuracy within and across our long-term care datasets. S2 additionally contains Figures S1a to S1m, showing the correlation and agreement between mass and volume estimates for determining nutritional intake at the whole plate level across all imaged samples. The left panel depicts the goodness of fit with linear regression and coefficient of determination (r^2^), and the right panel depicts the degree of agreement between measures and bias from the Bland-Altman method.

## Abbreviations

AFINI-T: automated food imaging and nutrient intake tracking
CNN: convolutional neural network
EDFN: encoder-decoder food network
IOU: intersection over union
LTC: long-term care
MTF: modified texture food
RD: registered dietitian
RGB-D: red-green-blue-depth
RTF: regular texture food
UNIMIB+: UNIMIB2016 with additional green representation

## Data Availability

The data sets can be made available upon reasonable request.
