# Supplementary material for: Enhancing Food Intake Tracking in Long-term Care With Automated Food Imaging and Nutrient Intake Tracking (AFINI-T) Technology: Validation and Feasibility Assessment
Source: JMIR Aging. 2022 Nov 17;5(4):e37590. doi: 10.2196/37590 (PMC9716425; doi:10.2196/37590)
Supplement: Multimedia Appendix 1 [file aging_v5i4e37590_app1.pdf]

Multimedia Appendix

S1. Standardizing Nutrient Values

Table S1 describes the workflow in converting % daily values to absolute measurements.

Table S1: Converting the regular texture dataset % daily values into absolute value to match the modified texture dataset. The Canadian Recommended Dietary Allowance (RDA) or Adequate Intake (AI) was used for individuals over 70 years of age and an average across the two sexes was assumed to represent 100% daily values [20].

	RDA/AI for > 70 Years			
**Nutrient (Input Units)**	**Males**	**Females**	**Assumed 100% Daily Value**	**Output Units**
Fat (g)	n/a	n/a	n/a	g
Carbohydrates (g)	n/a	n/a	n/a	g
Fibre (g)	n/a	n/a	n/a	g
Protein (g)	n/a	n/a	n/a	g
Calcium%	1200 mg	1200 mg	1200 mg	mg
Iron%	8 mg	8 mg	8 mg	mg
Sodium (mg)	n/a	n/a	n/a	mg
Vitamin B6%	1.7 mg	1.5 mg	1.6 mg	mg
Vitamin C%	90 mg	75 mg	82.5 mg	mg
Vitamin D (IU)	ns	ns	ns	IU
Vitamin K (mcg)	ns	ns	ns	mcg
Zinc (%)	11 mg	8 mg	9.5 mg	mg

Where: g: grams, mg: milligrams, IU: international units, mcg: micrograms. While the AI is known for vitamins D and K, no foods in the regular texture foods dataset reported % daily values and are thus marked as not specified (ns)

S2. Nutrient Intake Accuracies

Tables S2, S3,and S4 provide a comprehensive overview of nutrient intake accuracy within and across our LTC datasets. For each of the following nutrients of interest, Figures S1a to S1m show the correlation and agreement between mass and volume estimates methods for determining nutritional intake at the whole plate level across all imaged samples. The left panel depicts the goodness of fit with linear regression and coefficient of determination (r^2), right depicts the degree of agreement between measures and bias from the Bland-Altman method.

Table S2: Macronutrient intake accuracies within and across datasets.

**Dataset**		**Nutrient intake accuracy** $\mu \pm \sigma$ **(% error)**				
**Meal**	**# of classes (# images)**	**Calories (kcal)**	**Carbohydrates (g)**	**Fibre (g)**	**Fats (g)**	**Protein (g)**
RTD: B	3 (125)	18.13 ± 16.76 (18)	2.68 ± 2.36 (3)	0.50 ± 0.48 (1)	0.67 ± 0.74 (1)	1.08 ± 1.07 (1)
RTD: L	3 (125)	23.43 ± 21.64 (23)	2.70 ± 2.99 (3)	0.47 ± 0.30 (0)	0.88 ± 0.82 (1)	0.82 ± 0.85 (1)
RTD: D	3 (125)	17.04 ± 17.38 (17)	2.57 ± 2.37 (3)	0.22 ± 0.23 (0)	0.66 ± 0.80 (1)	0.59 ± 0.75 (1)
RTF subtotal	*9 (375)*	*16.31 ± 13.63 (16)*	*2.11 ± 2.05 (2)*	*0.37 ± 0.39 (0)*	*0.83 ± 0.80 (1)*	*1.11 ± 0.98 (1)*
MTD: D1 - L	15 (90)	9.31 ± 13.27 (9)	1.38 ± 1.52 (1)	0.08 ± 0.07 (0)	0.28 ± 0.57 (0)	0.31 ± 0.52 (0)
MTD: D1 - D	5 (25)	28.17 ± 25.01 (28)	1.33 ± 1.06 (1)	0.09 ± 0.07 (0)	1.19 ± 1.09 (1)	2.93 ± 2.69 (3)
MTD: D2- L	12 (74)	n/a	n/a	n/a	n/a	n/a
MTD: D2 - D	12 (90)	37.25 ± 38.66 (37)	3.26 ± 4.10 (3)	0.11 ± 0.14 (0)	1.92 ± 1.83 (2)	1.77 ± 1.57 (2)
MTD: D3 - L	10 (85)	12.46 ± 9.61 (12)	1.41 ± 1.15 (1)	0.13 ± 0.09 (0)	0.43 ± 0.33 (0)	0.72 ± 0.52 (1)
MTD: D3 - D	15 (109)	5.06 ± 3.53 (5)	1.30 ± 0.90 (1)	0.10 ± 0.07 (0)	0.02 ± 0.01 (0)	0.05 ± 0.03 (0)
MTD: D4 - L	9 (60)	5.90 ± 3.55 (6)	0.35 ± 0.16 (0)	0.08 ± 0.06 (0)	0.46 ± 0.32 (0)	0.14 ± 0.10 (0)
MTD: D4 - D	10 (90)	5.06 ± 5.41 (5)	0.81 ± 0.86 (1)	0.23 ± 0.25 (0)	0.18 ± 0.20 (0)	0.19 ± 0.20 (0)
MTD: D5 - L	5 (41)	14.98 ± 8.52 (15)	2.11 ± 1.21 (2)	0.19 ± 0.11 (0)	0.44 ± 0.26 (0)	0.65 ± 0.36 (1)
MTD subtotal	*93 (664)*	*18.44 ± 23.82 (18)*	*1.72 ± 2.17 (2)*	*0.13 ± 0.13 (0)*	*0.78 ± 1.14 (1)*	*1.14 ± 1.70 (1)*
**TOTAL**	**104 (1039)**	**16.85 ± 16.83 (17)**	**2.01 ± 2.09 (2)**	**0.31 ± 0.36 (0)**	**0.82 ± 0.90 (1)**	**1.12 ± 1.20 (1)**

There were no samples for Day 5 Dinner and no recipes available for foods imaged on Day 2 Lunch.
RTF: Regular Texture Foods Dataset; MTD: Modified Texture Foods Dataset; B: Breakfast, L: Lunch, D: Dinner; D#: Day number. For example, MTD: D1 - L is Modified Texture Foods Dataset: Day 1 - Lunch.

Table S3: Micronutrient intake accuracies of elements within and across datasets.

**Dataset**		**Nutrient intake accuracy** $\mu \pm \sigma$ **(% error)**			
**Meal**	**# of classes (# images)**	**Calcium (mg)**	**Iron (mg)**	**Sodium (mg)**	**Zinc (mg)**
RTD: B	3 (125)	10.66 ± 10.09 (11)	0.26 ± 0.24 (0)	20.77 ± 18.60 (21)	0.04 ± 0.03 (0)
RTD: L	3 (125)	11.96 ± 12.34 (12)	0.11 ± 0.07 (0)	32.20 ± 34.33 (32)	0.00 ± 0.00 (0)
RTD: D	3 (125)	2.95 ± 3.72 (3)	0.08 ± 0.09 (0)	45.71 ± 46.31 (46)	0.00 ± 0.00 (0)
RTF subtotal	*9 (375)*	*9.94 ± 8.48 (10)*	*0.17 ± 0.18 (0)*	*18.50 ± 15.58 (18)*	*0.05 ± 0.06 (0)*
MTD: D1 - L	15 (90)	8.45 ± 14.01 (8)	0.05 ± 0.05 (0)	16.43 ± 20.69 (16)	0.02 ± 0.02 (0)
MTD: D1 - D	5 (25)	1.86 ± 1.64 (2)	0.62 ± 0.57 (1)	17.05 ± 15.11 (17)	0.50 ± 0.46 (1)
MTD: D2- L	12 (74)	n/a	n/a	n/a	n/a
MTD: D2 - D	12 (90)	6.04 ± 7.03 (6)	0.17 ± 0.19 (0)	11.16 ± 9.22 (11)	0.10 ± 0.12 (0)
MTD: D3 - L	10 (85)	2.53 ± 2.03 (3)	0.09 ± 0.08 (0)	13.87 ± 11.42 (14)	0.05 ± 0.04 (0)
MTD: D3 - D	15 (109)	1.08 ± 0.75 (1)	0.02 ± 0.01 (0)	0.08 ± 0.06 (0)	0.01 ± 0.01 (0)
MTD: D4 - L	9 (60)	1.34 ± 0.93 (1)	0.03 ± 0.02 (0)	12.11 ± 8.05 (12)	0.01 ± 0.01 (0)
MTD: D4 - D	10 (90)	2.98 ± 3.22 (3)	0.07 ± 0.08 (0)	3.84 ± 3.32 (4)	0.04 ± 0.04 (0)
MTD: D5 - L	5 (41)	7.12 ± 4.54 (7)	0.12 ± 0.06 (0)	18.93 ± 10.33 (19)	0.10 ± 0.06 (0)
MTD subtotal	*93 (664)*	*4.37 ± 6.19 (4)*	*0.19 ± 0.34 (0)*	*12.92 ± 12.73 (13)*	*0.14 ± 0.27 (0)*
**TOTAL**	**104 (1039)**	**8.51 ± 8.31 (9)**	**0.18 ± 0.23 (0)**	**17.07 ± 15.09 (17)**	**0.07 ± 0.15 (0)**

There were no samples for Day 5 Dinner and no recipes available for foods imaged on Day 2 Lunch.
RTD: Regular texture foods dataset; MTD: Modified texture foods dataset; B: Breakfast, L: Lunch, D: Dinner; D#: Day number. For example, MTD: D1 - L is Modified Texture Foods Dataset: Day 1 - Lunch.

Table S4: Micronutrient intake accuracies of vitamins within and across datasets.

**Dataset**		**Nutrient intake accuracy** $\mu \pm \sigma$ **(% error)**			
**Meal**	**# of classes (# images)**	**Vitamin B6 (mg)**	**Vitamin C (mg)**	**Vitamin D (IU)**	**Vitamin K (mcg)**
RTD: B	3 (125)	0.02 ± 0.02 (0)	0.00 ± 0.00 (0)	n/s	n/s
RTD: L	3 (125)	0.00 ± 0.00 (0)	5.70 ± 4.05 (6)	n/s	n/s
RTD: D	3 (125)	0.00 ± 0.00 (0)	0.78 ± 0.96 (1)	n/s	n/s
RTF subtotal	*9 (375)*	*0.01 ± 0.02 (0)*	*0.00 ± 0.00 (0)*	*n/s*	*n/s*
MTD: D1 - L	15 (90)	0.01 ± 0.01 (0)	1.87 ± 1.59 (2)	0.00 ± 0.01 (0)	2.02 ± 2.29 (2)
MTD: D1 - D	5 (25)	0.10 ± 0.09 (0)	0.71 ± 0.50 (1)	4.38 ± 4.03 (4)	0.32 ± 0.29 (0)
MTD: D2- L	12 (74)	n/a	n/a	n/a	n/a
MTD: D2 - D	12 (90)	0.02 ± 0.03 (0)	0.18 ± 0.19 (0)	0.06 ± 0.09 (0)	0.01 ± 0.01 (0)
MTD: D3 - L	10 (85)	0.01 ± 0.00 (0)	0.31 ± 0.21 (0)	0.00 ± 0.00 (0)	1.69 ± 1.18 (2)
MTD: D3 - D	15 (109)	0.01 ± 0.01 (0)	1.70 ± 1.18 (2)	0.00 ± 0.00 (0)	0.16 ± 0.11 (0)
MTD: D4 - L	9 (60)	0.00 ± 0.00 (0)	0.66 ± 0.46 (1)	0.00 ± 0.00 (0)	2.33 ± 1.63 (2)
MTD: D4 - D	10 (90)	0.01 ± 0.01 (0)	2.80 ± 3.04 (3)	0.03 ± 0.03 (0)	3.55 ± 3.86 (4)
MTD: D5 - L	5 (41)	0.01 ± 0.01 (0)	0.96 ± 0.70 (1)	0.16 ± 0.10 (0)	0.66 ± 0.36 (1)
MTD subtotal	*93 (664)*	*0.03 ± 0.05 (0)*	*1.05 ± 1.50 (1)*	*0.87 ± 2.41 (1)*	*1.13 ± 1.98 (1)*
**TOTAL**	**104 (1039)**	**0.02 ± 0.03 (0)**	**0.27 ± 0.88 (0)**	**0.87 ± 2.41 (1)**	**1.13 ± 1.98 (1)**

There were no samples for Day 5 Dinner and no recipes available for foods imaged on Day 2 Lunch. RTF: Regular Texture Foods Dataset; MTD: Modified Texture Foods Dataset; B: Breakfast, L: Lunch, D: Dinner; D#: Day number. For example, MTD: D1 - L is Modified Texture Foods Dataset: Day 1 - Lunch.

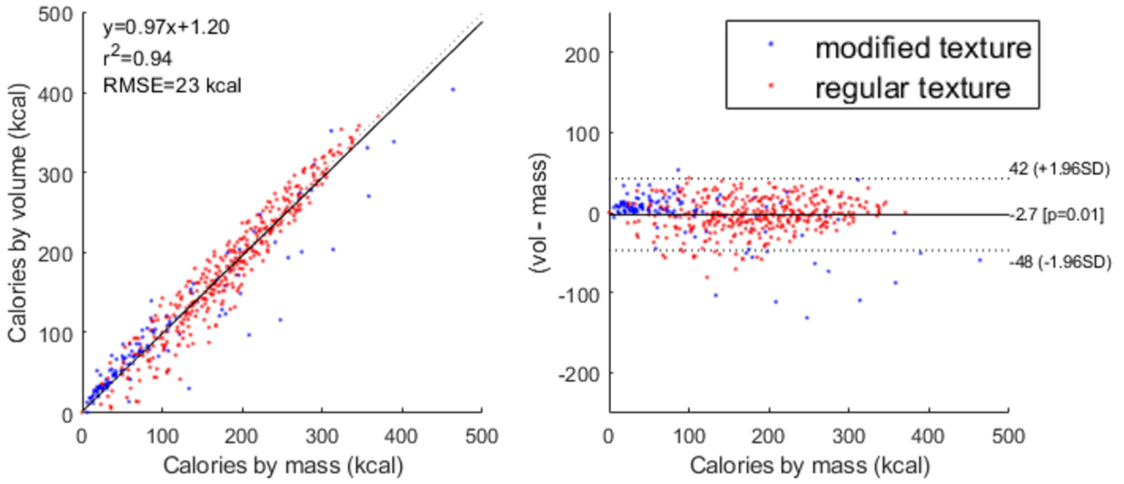

(a) Correlation and agreement between mass and volume estimates calories.

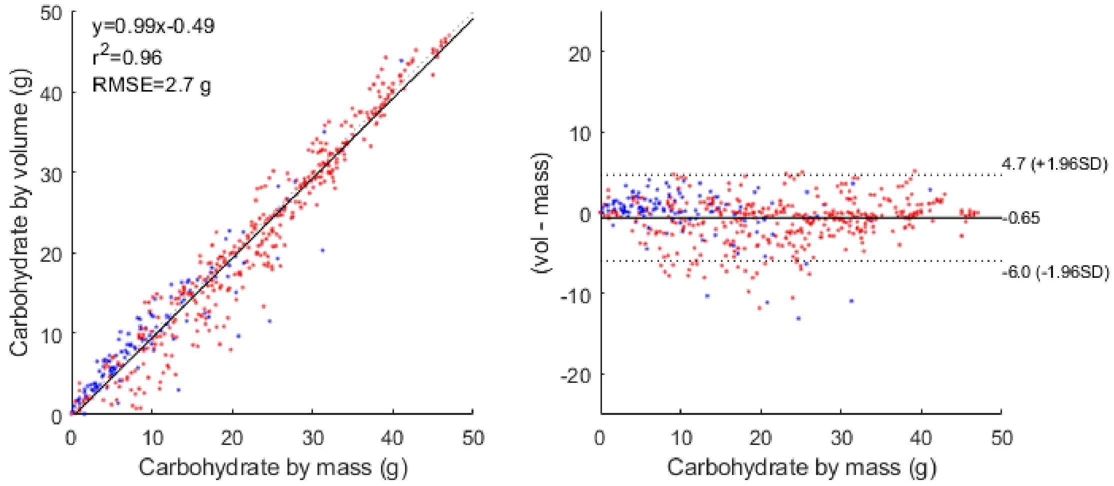

(b) Correlation and agreement between mass and volume estimates of carbohydrates.

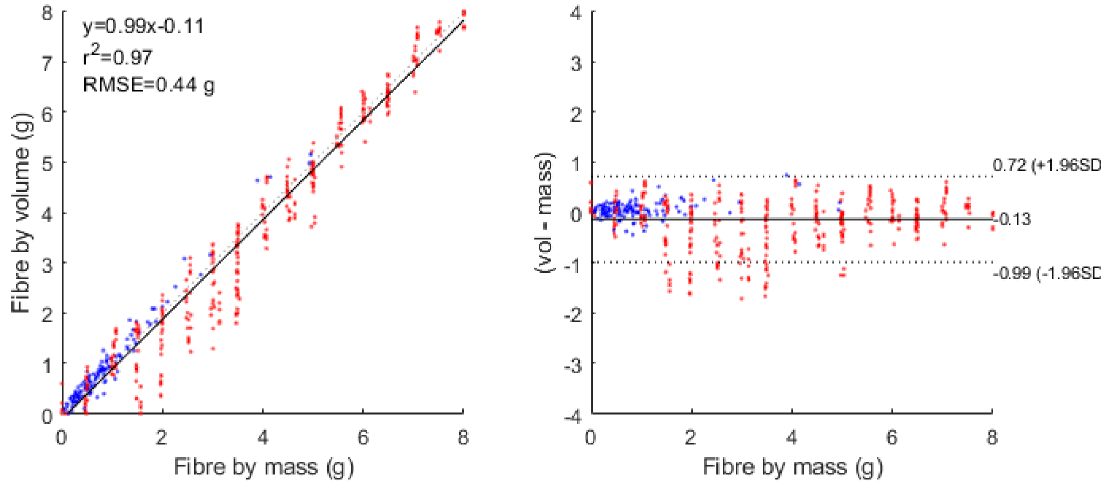

(c) Correlation and agreement between mass and volume estimates of fibre.

Figure S1: Correlation and agreement between mass and volume nutrient estimates.

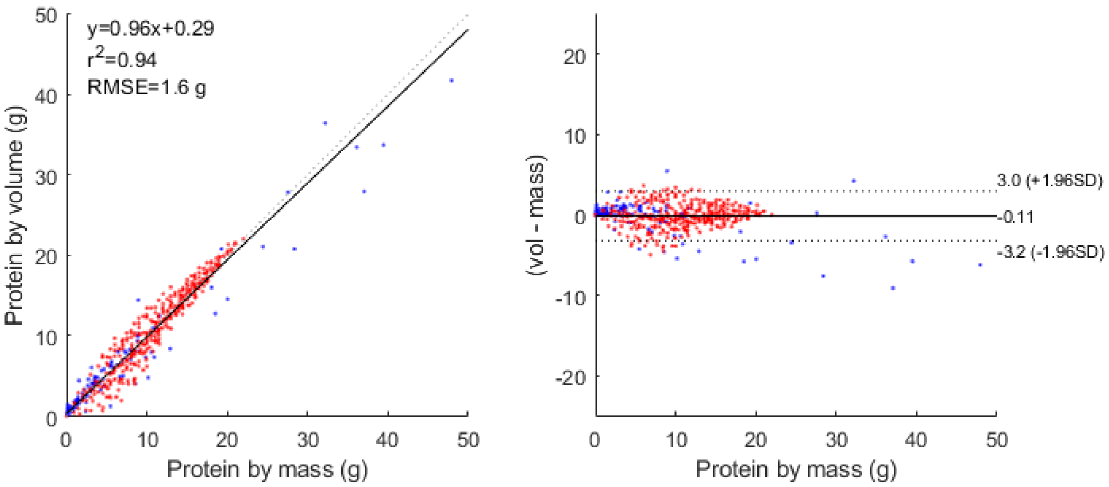

(d) Correlation and agreement between mass and volume estimates of protein.

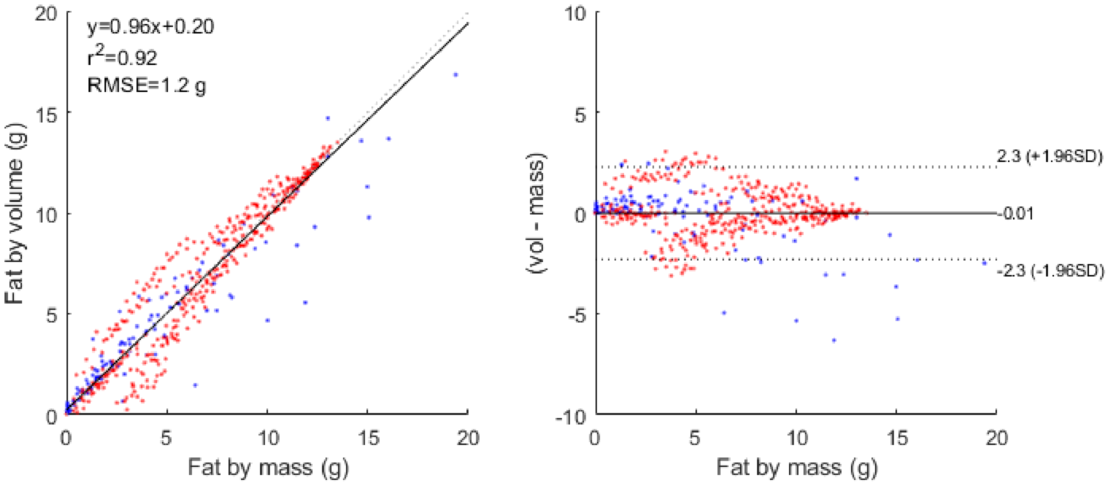

(e) Correlation and agreement between mass and volume estimates of fat.

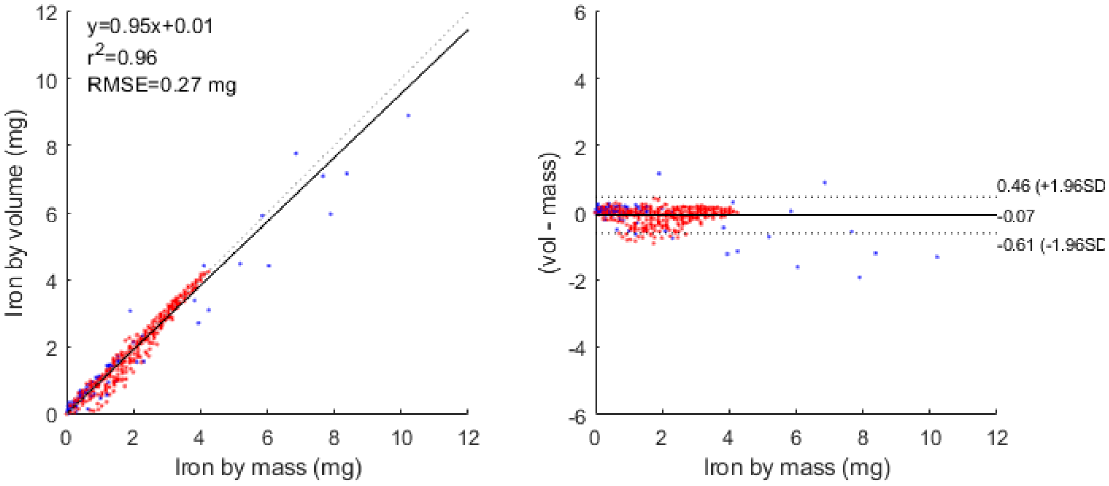

(f) Correlation and agreement between mass and volume estimates of iron.

Figure S1: Continued correlation and agreement between mass and volume nutrient estimates.

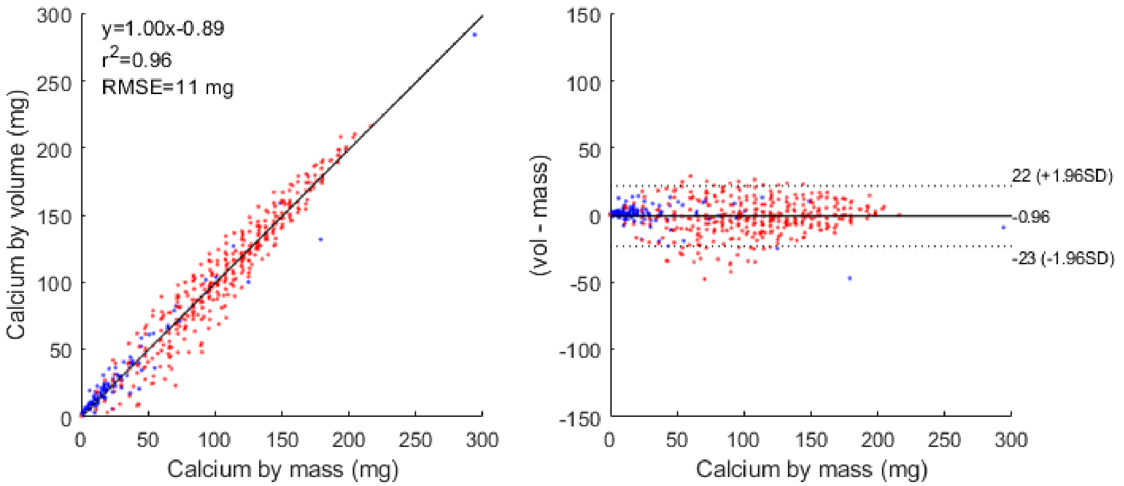

(g) Correlation and agreement between mass and volume estimates of calcium.

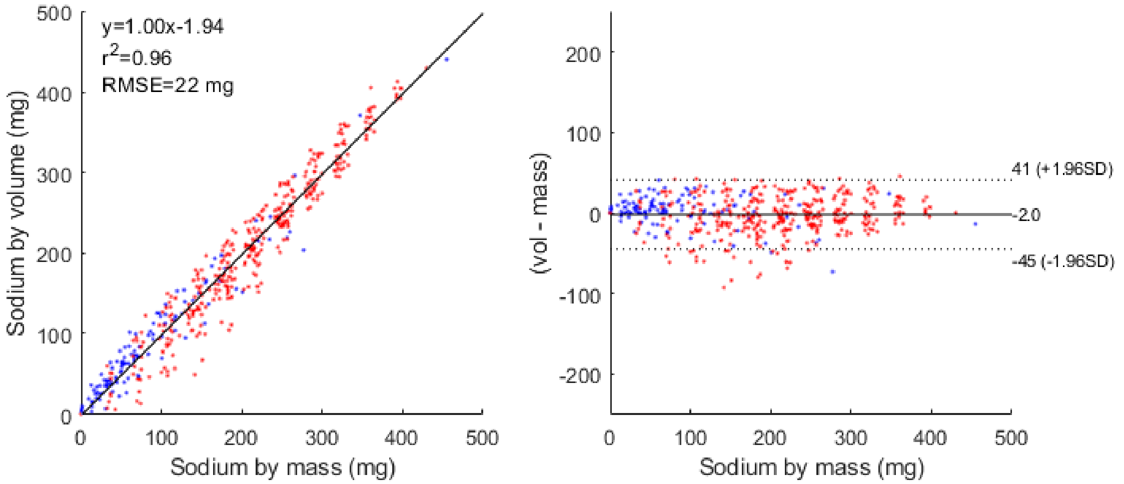

(h) Correlation and agreement between mass and volume estimates of sodium.

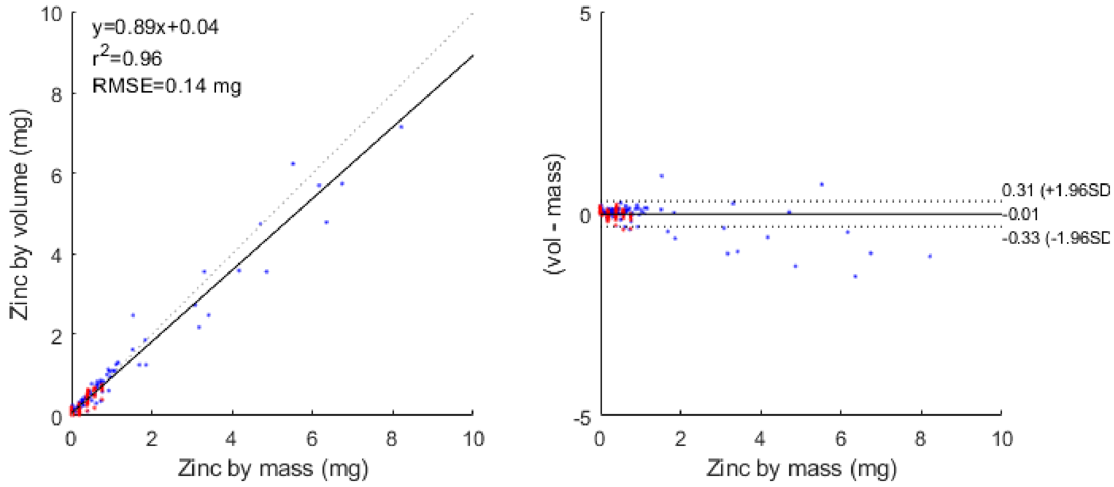

(i) Correlation and agreement between mass and volume estimates of zinc.

Figure S1: Continued correlation and agreement between mass and volume nutrient estimates.

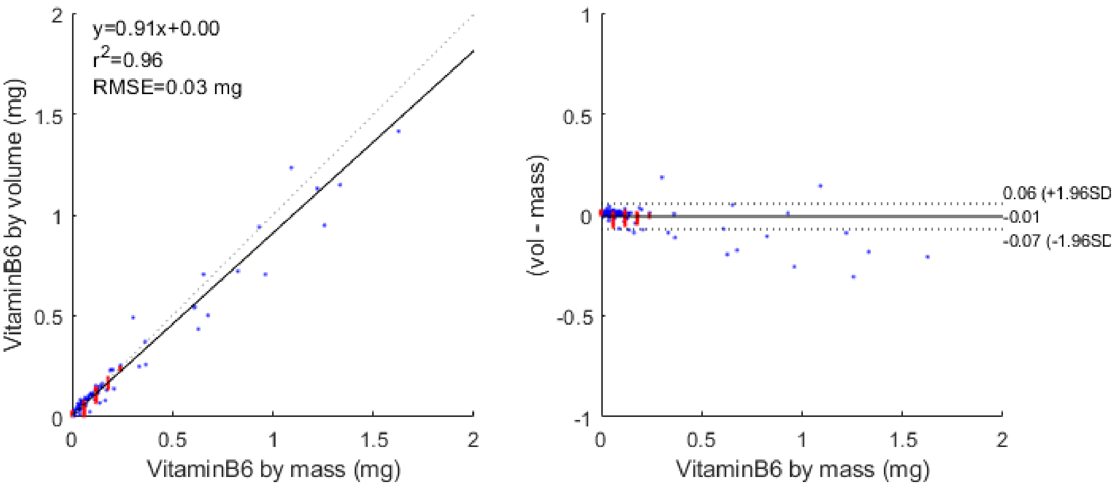

(j) Correlation and agreement between mass and volume estimates of vitamin B6.

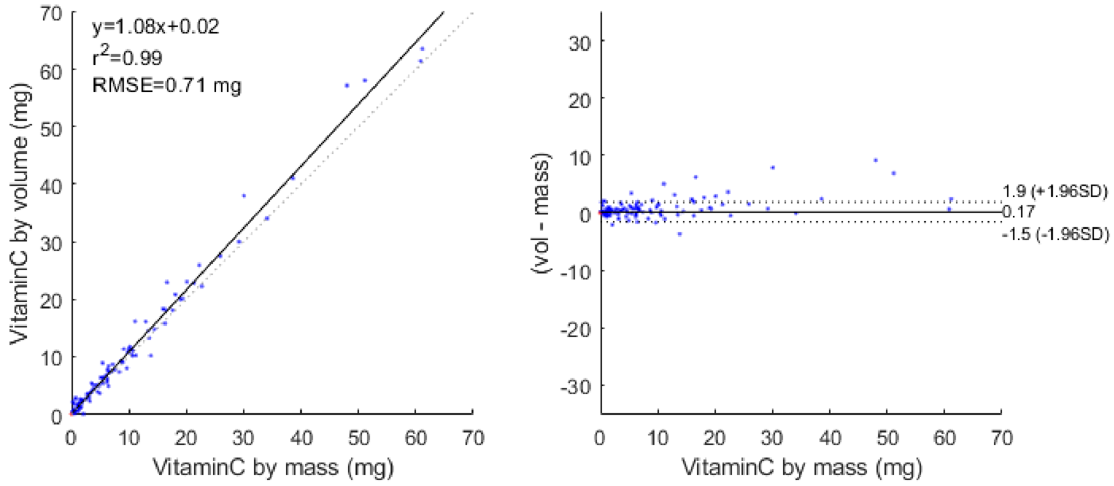

(k) Correlation and agreement between mass and volume estimates of vitamin C.

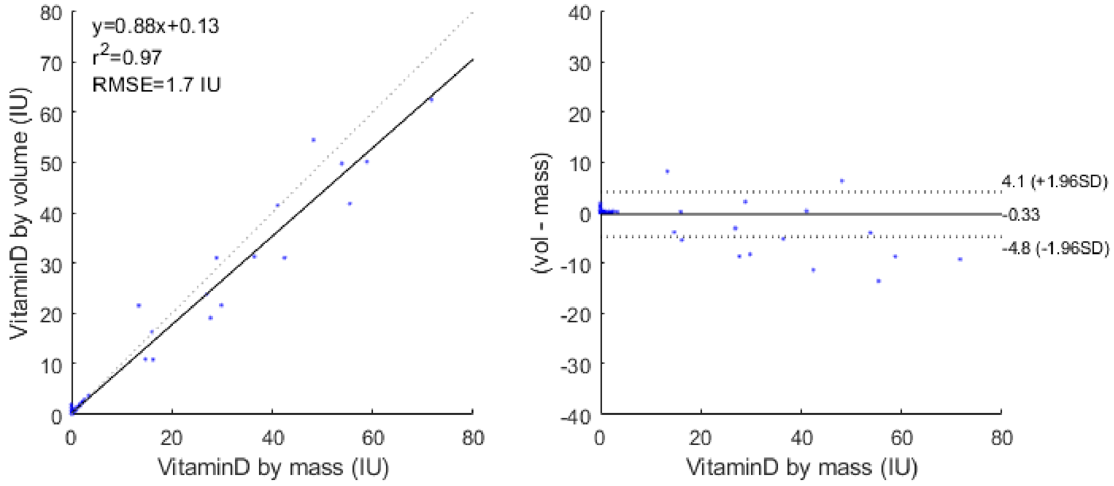

(l) Correlation and agreement between mass and volume estimates of vitamin D.

Figure S1: Continued correlation and agreement between mass and volume nutrient estimates.

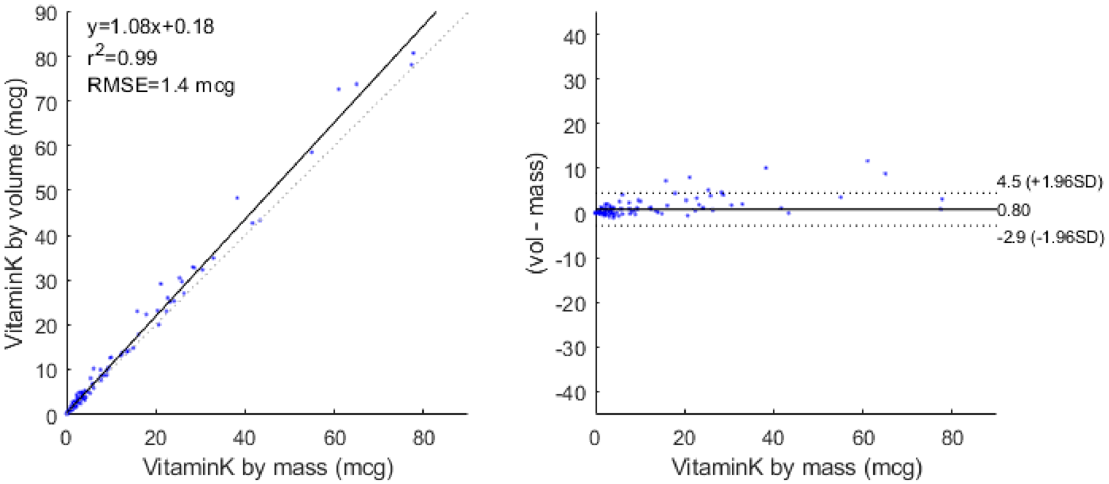

(m) Correlation and agreement between mass and volume estimates of vitamin K.

Figure S1: Continued correlation and agreement between mass and volume nutrient estimates.

References
